# EMILIN proteins are novel extracellular constituents of the dentin-pulp complex

**DOI:** 10.1038/s41598-020-72123-2

**Published:** 2020-09-18

**Authors:** Thomas Imhof, Yüksel Korkmaz, Manuel Koch, Gerhard Sengle, Alvise Schiavinato

**Affiliations:** 1 Center for Biochemistry, Medical Faculty, University of Cologne, 50931 Cologne, Germany; 2 Institute for Dental Research and Oral Musculoskeletal Biology, Medical Faculty, University of Cologne, 50931 Cologne, Germany; 3 Department of Periodontology and Operative Dentistry, University Medical Center of the Johannes Gutenberg University, 55131 Mainz, Germany; 4 Department of Pediatrics and Adolescent Medicine, Faculty of Medicine and University Hospital Cologne, University of Cologne, 50931 Cologne, Germany; 5 Center for Molecular Medicine Cologne (CMMC), University of Cologne, 50931 Cologne, Germany; 6 Cologne Center for Musculoskeletal Biomechanics (CCMB), 50931 Cologne, Germany

**Keywords:** Dental pulp, Cell biology

## Abstract

Odontoblasts and pulp stroma cells are embedded within supramolecular networks of extracellular matrix (ECM). Fibrillin microfibrils and associated proteins are crucial constituents of these networks, serving as contextual scaffolds to regulate tissue development and homeostasis by providing both structural and mechanical properties and sequestering growth factors of the TGF-β superfamily. EMILIN-1, -2, and -3 are microfibril-associated glycoproteins known to modulate cell behaviour, growth factor activity, and ECM assembly. So far their expression in the various cells of the dentin-pulp complex during development, in the adult stage, and during inflammation has not been investigated. Confocal immunofluorescence microscopy and western blot analysis of developing and adult mouse molars and incisors revealed an abundant presence of EMILINs in the entire dental papilla, at early developmental stages. Later in development the signal intensity for EMILIN-3 decreases, while EMILIN-1 and -2 staining appears to increase in the pre-dentin and in the ECM surrounding odontoblasts. Our data also demonstrate new specific interactions of EMILINs with fibulins in the dentin enamel junction. Interestingly, in dentin caries lesions the signal for EMILIN-3 was significantly increased in inflamed odontoblasts. Overall our findings point for the first time to a role of EMILINs in dentinogenesis, pulp biology, and inflammation.

## Introduction

Differentiation of ameloblasts and odontoblasts is controlled by an extensive epithelial-mesenchymal crosstalk^1^. Extracellular matrix (ECM) proteins have specific functions in this context since they not only provide the substrate for ameloblast adhesion^2^, but also facilitate proper enamel and dentin formation and mineralization^3,4^. First, the enamel epithelium and the pulp mesenchyme are separated by a basement membrane (BM). Upon mineralisation this structure is processed and is therefore called dentin enamel junction (DEJ). In newborn mice the mineralisation has just started in the cusp of the crown and a BM is still found in the basal crown. The dentin enamel junction is essential for the stable dentin enamel attachment and contains BM proteins throughout postnatal life^5^. After completion of the secondary dentition, humans lose the ability to form enamel. However, the human pulp still possesses a limited regeneration potential to activate pulp progenitor cells and to form tertiary dentin^6^. This regenerative capacity is achieved by the sophisticated interplay between stem cells such as dental pulp stem cells (DPSCs) and their ECM microenvironment^7^. Understanding the complex processes by which the structural integrity of the ECM controls stem cell behaviour opens novel therapeutic avenues for dental pulp replacement strategies using tissue engineering^8,9^. In recent years our view of the ECM has changed from considering it a passive structural scaffold to seeing it as a dynamic network required for the regulation of tissue and organ development and for maintenance of homeostasis^10^. The structural complexity of the ECM gives rise to unique tissue specific architectures, which function as informative cellular microenvironments^11^. To facilitate cellular communication, ECM networks regulate the bioavailability of growth factors in the connective tissue space. One of the best characterized ECM networks with this function is formed by fibrillin microfibrils (FMF), which facilitate extracellular targeting and sequestration of growth factors of the TGF-β superfamily^12,13^. Members of this family, such as TGF-β and BMPs, are well known to exert key functions in the regulation of odontogenesis and odontoblast differentiation^14–16^. TGF-β is secreted as a large latent complex (LLC) covalently tethered by disulfide bonds to latent TGF-β binding proteins (LTBPs), which target TGF-β to specific sites within the FMF^17,18^. However, BMPs are directly targeted to FMF via interactions of their pro-domains with FMF^19,20^. In addition to their function as targeting platforms for growth factors FMF serve as a deposition scaffold for tropoelastin thereby giving rise to mature elastic fibres after lysyl oxidase (LOX) mediated cross-linking. LOX is targeted to the ECM via fibulin-4^21^, which belongs to a family of proteins that also interact with FMF^22^. The fibrillar and microfibrillar collagen components as well as the FMF of the dentin-pulp complex connective tissue has been identified since long^23,24^, however, until now information on the localization and function of FMF associated ligands is scarce. During DPSC differentiation and mineralization, fibulin-1, LOX, and LOX-like (LOXL) family members were found to be upregulated, whereas fibulin-2 and LOXL2 were downregulated^25,26^. LOXL2 protein administration to human DPSCs (hDPSC) decreased early differentiation and mineralization, suggesting that LOXL2 inhibition can promote hDPSC differentiation to odontoblasts^26^. Also during wound healing of human dental pulp, fibrillins and the TGF-β carrier LTBP-1 were found to be co-expressed^27^. However, comprehensive information about the functional interactions necessary for ECM assembly and growth factor regulation in the dental pulp is required to understand the molecular pathways responsible for malformations in patients with mutations in ECM proteins. For instance, mutations in the fibrillin genes (*FBN1*, *FBN2*) lead to craniofacial features (e.g. retrognathia, dolichocephaly, high palate) and also to dental deformities such as root deformity and pulp calcification^28^.

Little is known about the localization and functional interactions of regulators of growth factor activity in the dental pulp ECM. We recently identified EMILINs (Elastin-Microfibril-Interface-Located-proteINs) as novel constituents of skeletal tissues, where they are required for the ECM incorporation of the LOX-binding protein fibulin-4 in skeletal tissues^29,30^. EMILINs constitute a family that exert unique functions in the regulation of connective tissue growth factors. For example, EMILINs were found to influence pro-TGF-β processing^31,32^, EMILIN-2 modulates Wnt activity by directly binding Wnt1^33^, and EMILIN-3 regulates the bioavailability of Hedgehog ligands^34^. A functional role for EMILINs was also reported in culture of human periodontal ligament fibroblasts, where it was required for FMF deposition^35^. However, our previous in vivo and in vitro studies in skin showed that EMILIN-1 ECM incorporation depends on the presence of FMF and not vice versa^36^, illustrating tissue specific differences in ECM assembly and structure. Despite their important function to modulate the activity of growth factors known to be essential for odontoblast differentiation, mineralization and regeneration, no information about the spatio-temporal localization of EMILINs and their functional interactions in the dental pulp is known. Here, for the first time, we investigated the localization of EMILINs in the dentin-pulp complex of developing, adult, and carious teeth. Moreover, we defined novel functional interactions with the DEJ components fibulin-1 and -2 and demonstrated co-localization with BM remnants within the junction.

## Results

### EMILINs are assembled into abundant networks in dental papilla and pulp during development and perinatal period

**Figure 1:**
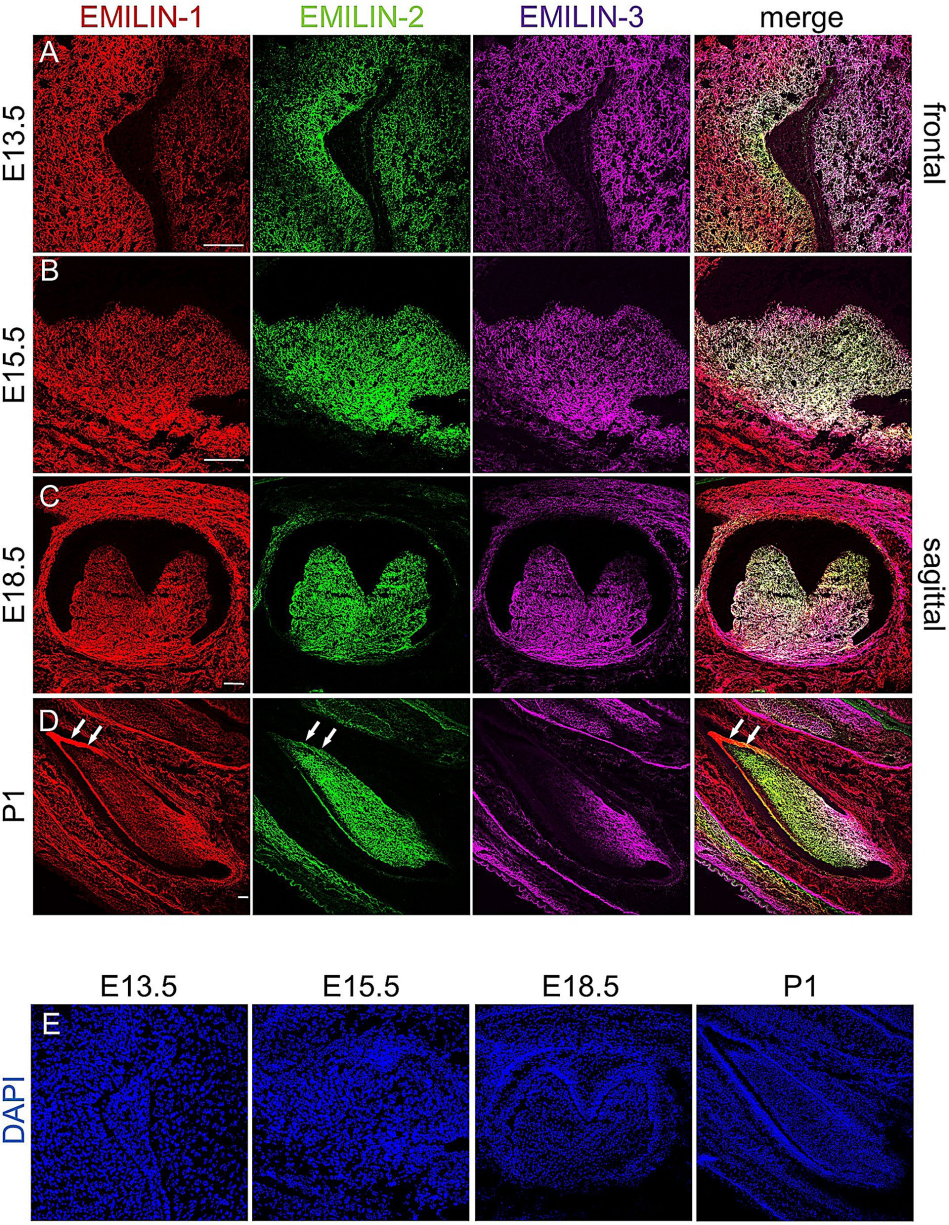
EMILIN localization at early embryonic stages of tooth development. (**A**) All three EMILINs are found in the condensed mesenchyme underlying the molar placode (E13.5 frontal section). (**B**,**C**) In E15.5 and 18.5 old embryos EMILIN-2 is concentrated in the molar papilla, while EMILIN-1 and -3 are also localized in the adjacent mesenchyme. EMILIN-1 and EMILIN-3 show a baso-coronal gradient in molars of E18.5 old embryos. (**D**) In the postnatal day 1 (P1) incisor EMILIN-1 and -2 are localized in the pulp and dentin enamel junction (white arrows), while EMILIN-3 staining is restricted to the basal pulp (**E**) DAPI counterstain for nuclei is shown. Scale bars, 50 μm.

**Figure 2:**
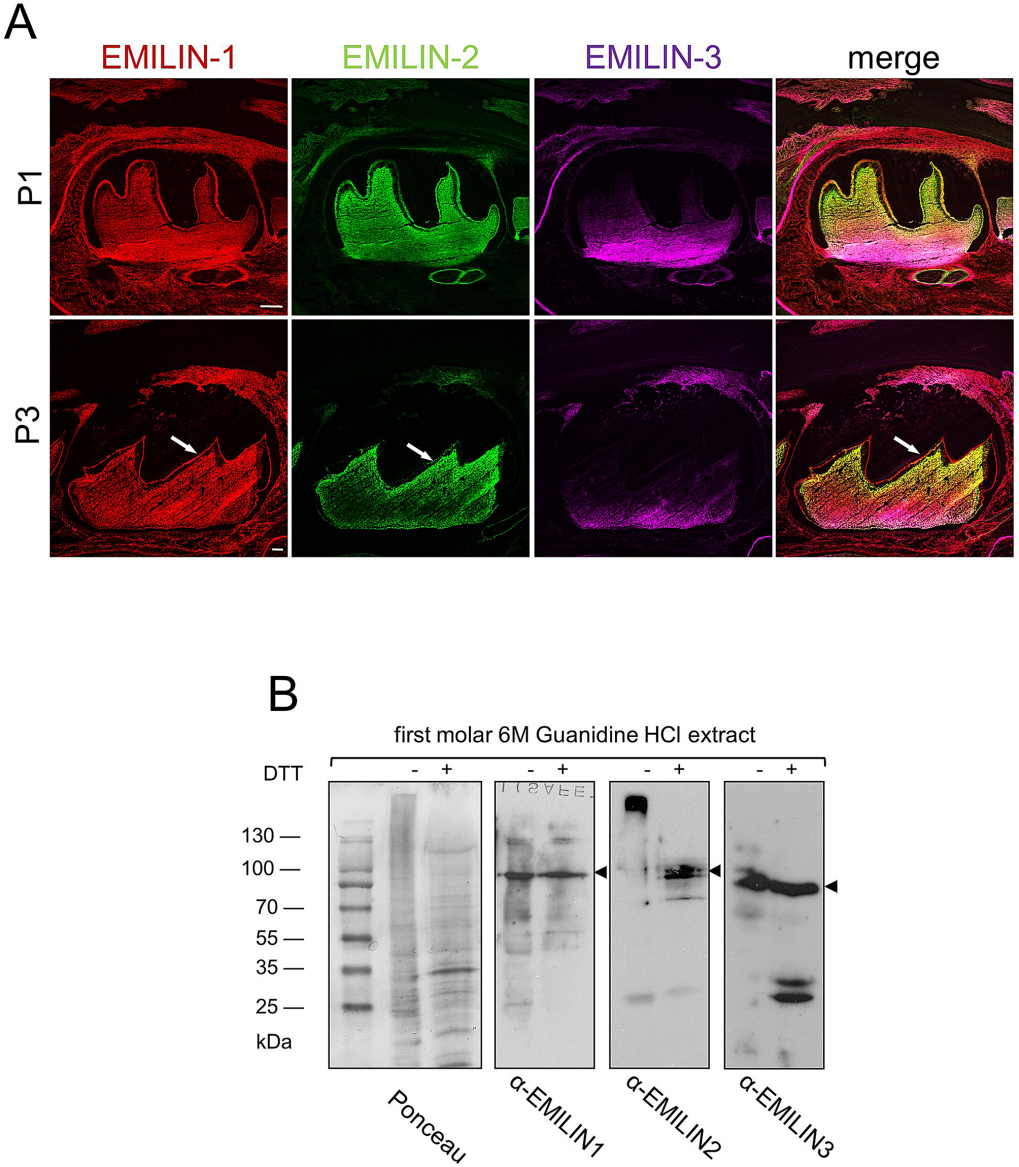
Early postnatal localization of EMILINs in molar pulp. (**A**) In newborn mice (postnatal day 1 and 3) EMILIN-1 and -2 are found throughout the molar pulp mesenchyme and within the dentin enamel junction (indicated by white arrows) separating ameloblasts from the odontoblasts. At the same stage, EMILIN-3 signal intensity shows a baso-coronal gradient without staining the dentin enamel junction. (**B**) The presence of all three EMILINs was verified by immunoblotting of first molar extracts. The three proteins appear at their corresponding molecular size (arrowheads)**.** Full-length blots are presented in Supplementary Fig. 4. Scale bars, 50 μm.

To study the localization of EMILINs we stained mouse teeth at different characteristic stages of tooth development. At embryonic day E13.5 positively stained EMILIN-1, -2, and -3 fibril networks were found in the condensed mesenchyme of molar dental buds. While EMILIN-1 and EMILIN-3 were also found in adjacent mesenchymal tissues, EMILIN-2 distribution was specifically restricted to the condensed mesenchyme of the dental papilla (Fig. 1A). In E15.5 and E18.5 embryos we found EMILIN-1 and -2 homogenously distributed in the entire molar dental papilla (Fig. 1B,C). At E15.5 and E18.5 the immunolocalization for EMILIN-2 was even more clearly restricted to the dental papilla, while the signal for EMILIN-1 and EMILIN-3 showed a baso-coronal gradient (Fig. 1B,C and Supplementary Fig. 1). In newborn incisors EMILIN-2 was evenly stained in the entire pulp mesenchyme, whereas EMILIN-1 and in particular EMILIN-3 were enriched in the region of the mesenchymal stem cell niche (Fig. 1D). Interestingly, EMILIN-1 and -2 were also found to be localized in the DEJ separating the ameloblasts of the inner enamel epithelium (IEE) from the underlying odontoblasts of the incisors dental pulp (Fig. 1D). In newborn molars EMILIN-1 and -2 were found throughout the whole pulp tissue and in the subodontoblastic region, whereas the localization of EMILIN-3 was restricted to the most basal part of the pulp (Fig. 2A). Notably, at this stage EMILIN-1 and -2 antibodies stained also the DEJ of molars. Protein presence of EMILINs was further confirmed by immunoblot analysis of molar extracts (Fig. 2B).

### EMILIN-1 and -2 localize to dentin enamel junction where they interact with fibulin-2

**Figure 3:**
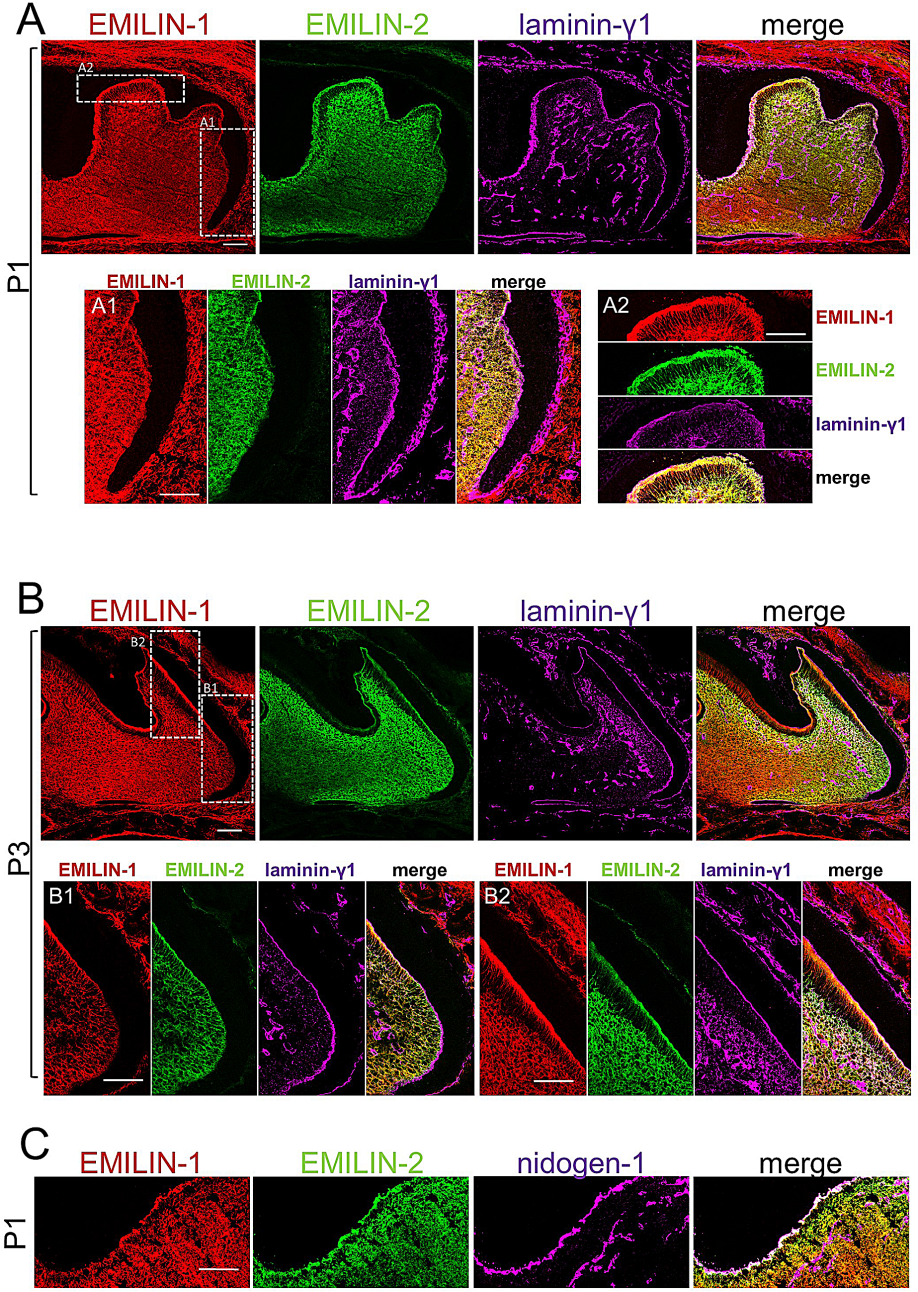
EMILIN localization within molar dentin enamel junction. Localization of EMILIN-1 and -2 within the dentin enamel junction was confirmed by co-labeling with laminin-γ1 (**A**,**B**) and nidogen-1 (**C**) in newborn (postnatal day 1 and 3) mouse molars. These stainings revealed weak signal intensity of EMILIN-1 and -2 within the basal epithelial-mesenchymal junction, which still resembles a genuine BM at P1. In contrast, at the coronal epithelial-mesenchymal junction both EMILIN-1 and -2 are strongly stained and co-localize with nidogen-1 and laminin-γ1. Scale bars, 50 μm.

**Figure 4:**
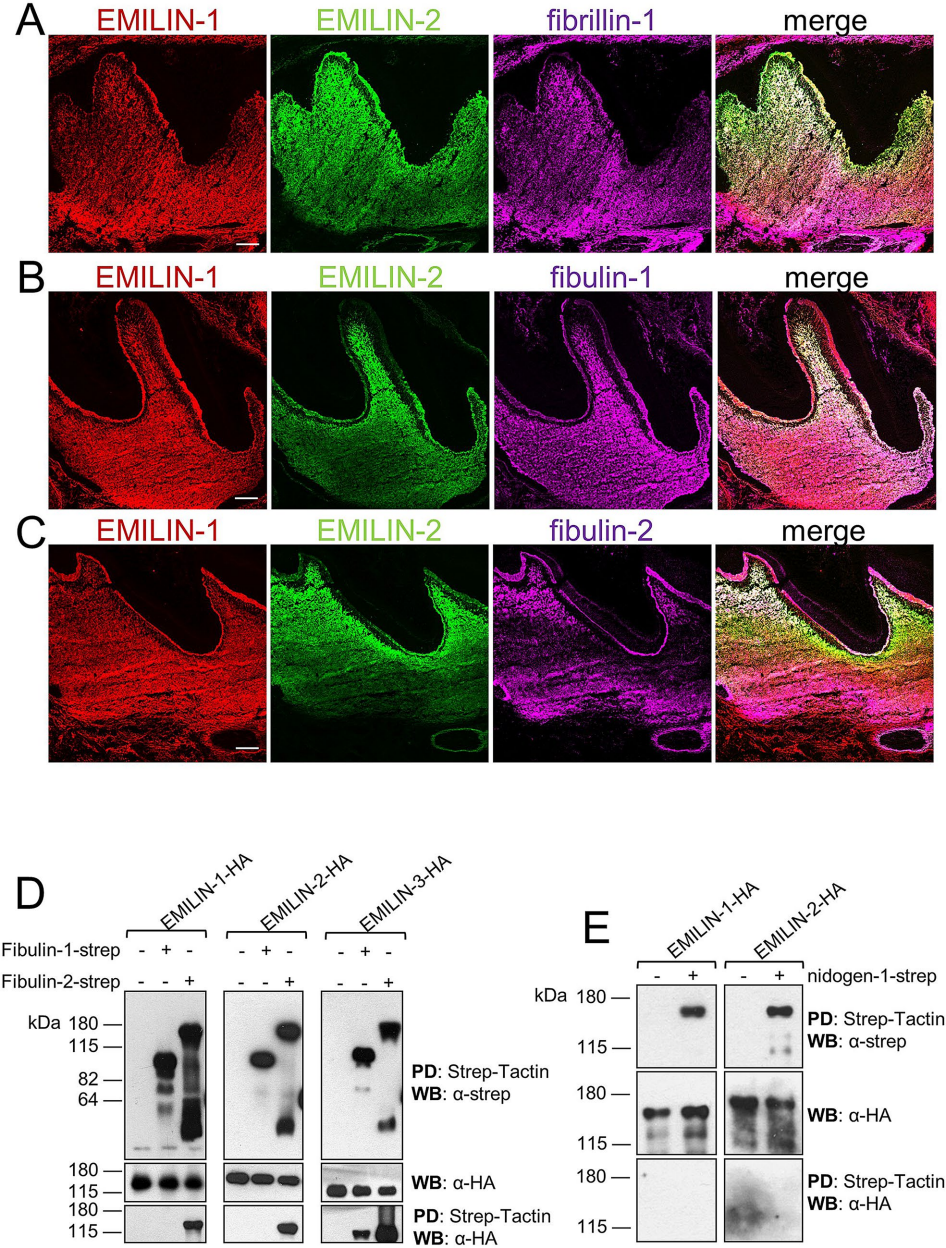
EMILIN co-localization and specific interactions with microfibril components in molars. EMILIN-1 and -2 were immunolabeled together with (**A**) fibrillin-1, (**B**) fibulin-1 and (**C**) fibulin-2 in newborn mouse molars. Fibrillin-1, fibulin-1 and -2 are co-localizing with EMILIN-1 and -2 in the pulp and in the dentin-enamel junction. (**D**) In vitro binding assays showing interaction of HA-tagged EMILINs with Strep-tagged fibulin-1 and -2 (**E**) but not with Strep-tagged nidogen-1. Top panel: blots for fibulins and nidogen-1 with an anti-Strep antibody after Strep-Tactin pull down; middle panel: EMILIN blots of supernatant before pull down; bottom panel: EMILIN blots after pull down. Full-length blots are presented in Supplementary Fig. 4. PD: pull down; WB: western blot. Scale bars, 50 μm.

To confirm and better characterize the distribution of EMILIN-1 and -2 at the DEJ, we performed co-localization studies with antibodies against the BM components laminin-γ1 and nidogen-1 (Fig. 3A–C). These stainings revealed that EMILIN-1 and -2 co-localize with BM proteins within the DEJ. Next, we also looked specifically for fibrillin-1 as we previously found that EMILINs are associated with fibrillin microfibrils in tissues^36,37^, and for fibulin-1 and -2 because these two fibulin family members are known components of the DEJ of developing teeth^38^. Co-localization studies confirmed the overlapping distribution of EMILIN-1 and -2 with all these markers in the pulp and within the DEJ (Fig. 4A–C). To biochemically test potential direct physical interactions between EMILINs and fibulins we performed in vitro binding assays using purified recombinant Strep-tagged fibulin-1 and -2 and supernatants from transiently transfected HEK293 cells overexpressing HA-tagged EMILINs. We found that EMILIN-1 and -2 interact with fibulin-2, while EMILIN-3 interacts with fibulin-1 and -2 (Fig. 4D). Both fibulin-1 and -2 were found to interact with elastin in a positive control experiment (Supplementary Fig. S2), while neither EMILIN-1 nor -2 bound the BM component nidogen-1 under similar experimental conditions (Fig. 4E).

### Localization of EMILINs to pulp mesenchyme, periodontal ligament and niches of specialized cells in adult molars and incisors

**Figure 5:**
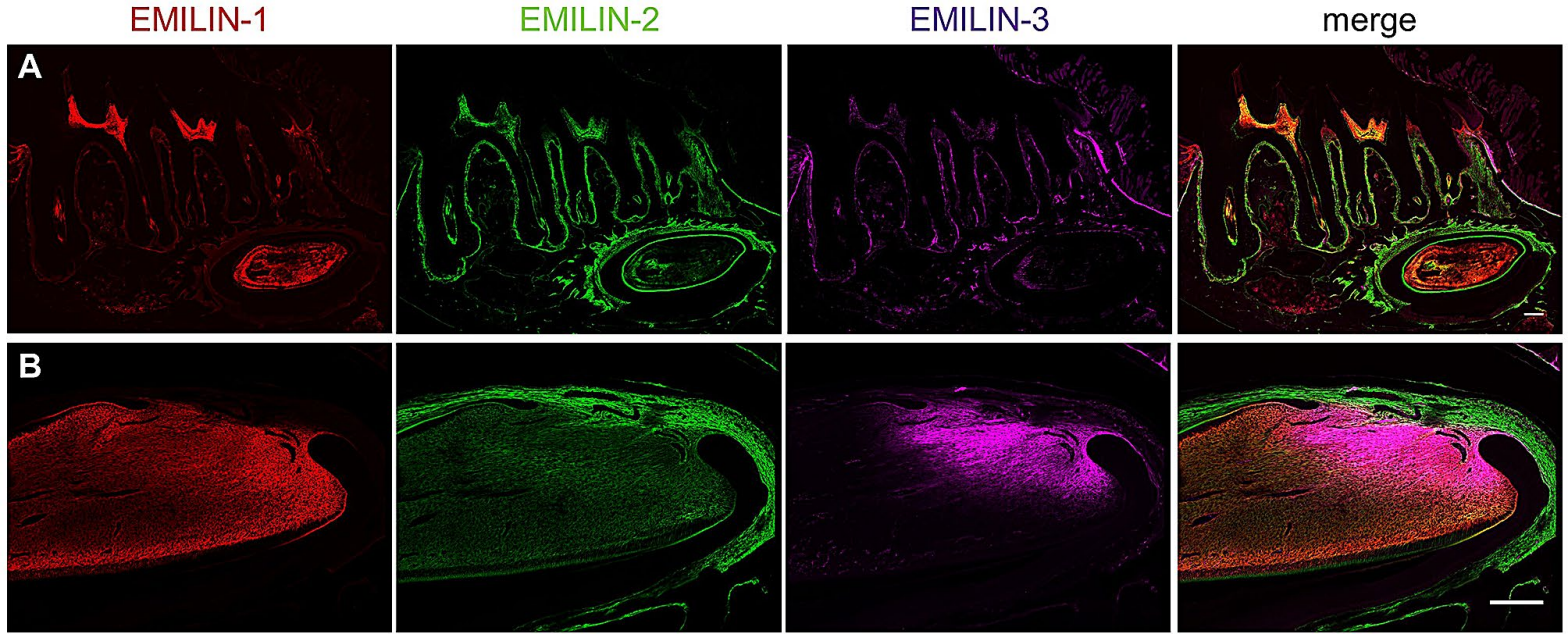
EMILIN localization in adult mice. In adult mice EMILIN-1 (red) is abundant in the molar (**A**) and incisor (**B**) pulp mesenchyme. EMILIN-2 (green) is localized in the molar and incisor pulp mesenchyme and in the periodontal ligament. EMILIN-3 (magenta) staining is found in the most apical pulp mesenchyme of molars and in the cementoblast cell layer. In the incisor EMILIN-3 is found in the region of the mesenchymal stem cell niche; here EMILIN-1 (red) and EMILIN-3 (magenta) are co-expressed. Scale bars, 500 µm (molars); 200 µm (incisors).

**Figure 6:**
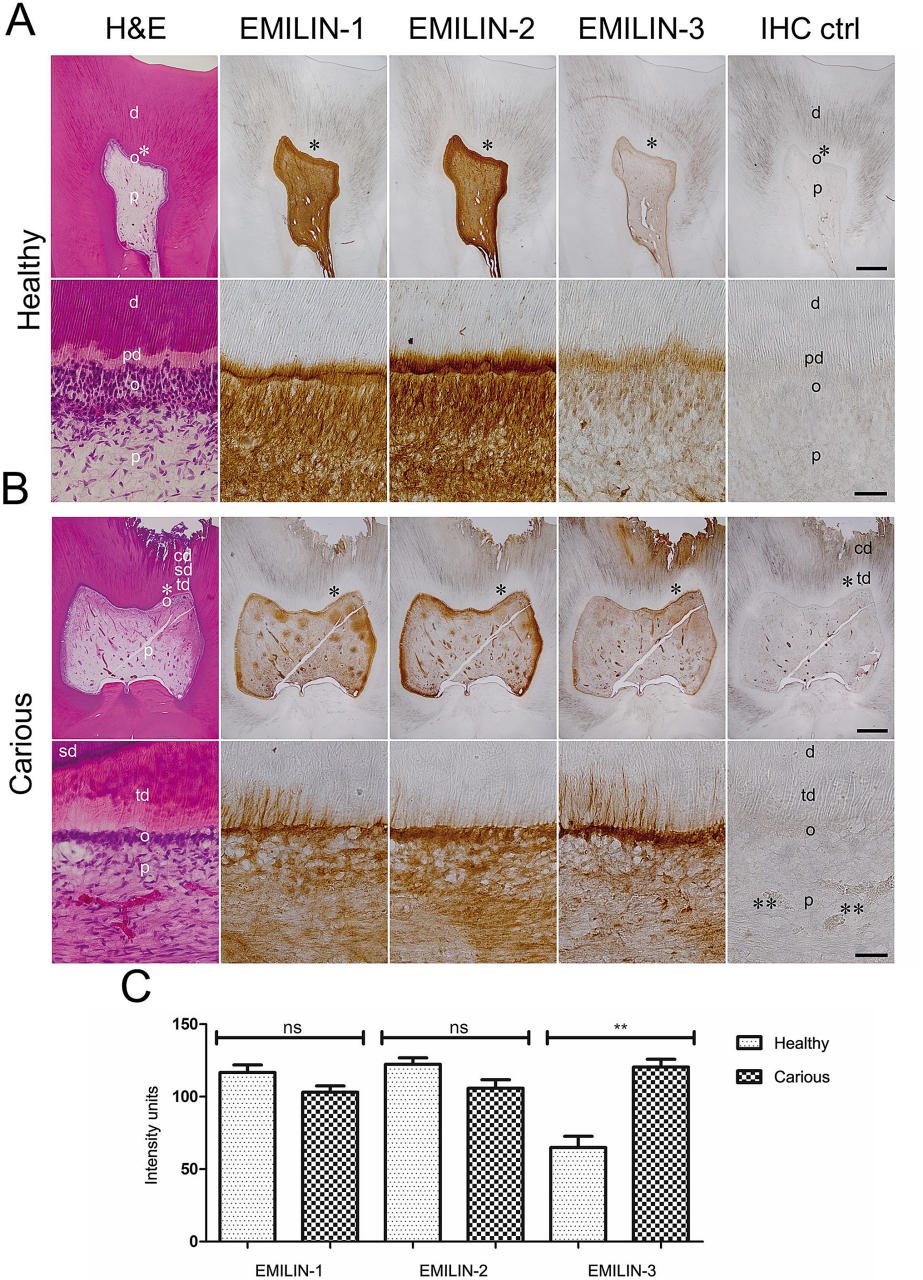
Localization of EMILINs in healthy and carious human molars. (**A**,**C**) Overview and detail images of consecutive sections of a healthy human molar show EMILIN-1 and -2 localization in the pre-dentin and in the odontoblast layer, while EMILIN-3 is only weakly detected in the odontoblast layer. (**B**,**C**) Overview and detail images of consecutive sections of dentin caries in an inflamed human molar, reveal a slightly decreased presence of EMILIN-1 and -2, while EMILIN-3 staining intensity is significantly upregulated in the odontoblasts underneath the caries lesion (*cd* caries defect, *d* dentin, *o* odontoblast, *p* pulp, *pd* pre-dentin, *sd* secondary dentin, *td* tertiary dentin). Single asterisks mark the magnified area. Double asterisks: mark a non-specific signal in blood vessels. IHC ctrl: as a control for the immunohistochemistry procedure primary antibodies were omitted. Scale bar overview, 1 mm; Scale bar detail, 50 µm.

Next, we analysed the localization of EMILINs in molars (Fig. 5A) and incisors (Fig. 5B) of adult mice: EMILIN-1 was found in the entire pulp mesenchyme of both molars and incisors, EMILIN-2 was localized in the entire pulp mesenchyme and in the periodontal ligament while EMILIN-3 was weakly stained in the periodontal ligament, but strongly and specifically localized in the presumptive incisor mesenchymal stem cell niche (Fig. 5A,B). In the healthy human dentin-pulp complex the ordered morphology of dentin (Fig. 6A), pre-dentin, odontoblasts, subodontoblastic regions, and dental pulp was assessed by H&E staining (Fig. 6A, upper image panel). In the consecutive sections of human molars, IHC signals for EMILIN-1 were detected in the odontoblast layer, in odontoblast processes within pre-dentin, and in the ECM of pre-dentin. Similarly, EMILIN-2 was detected in the odontoblast layer and in odontoblast processes within pre-dentin. The ECM of pre-dentin and the dental pulp also showed a strong staining for EMILIN-2. In contrast, weak EMILIN-3 signals were detected in a subpopulation of odontoblast cell bodies within the odontoblast layer. A faint EMILIN-3 signal was also present in the pre-dentin, while dental pulp ECM did not give any signal. Some spider-shaped cells in the pulp were positive for EMILIN-3 (Supplementary Fig. S3A) and co-immunofluorescence stainings with CD68 or HLA-DR antibodies marked the EMILIN-3 positive pulp cells (Supplementary Fig. S3B). Based on this finding, we hypothesize that the spider-shaped cells are either macrophages or dendritic cells.

### EMILIN-3 is significantly upregulated in dentin caries lesions

Diagnosis of dentin caries (Fig. 6B) was carried out by H&E staining revealing tertiary dentin regions with unordered dentin tubules underneath the caries cavity (Fig. 6B, lower image panel). In comparison to the healthy odontoblasts layer (Fig. 6A), also, the thickness of the odontoblast layer was reduced (the decrease of the number of odontoblasts) (Fig. 6B). In the dental pulp underneath the caries region, numerous inflammatory cells with acute and chronic inflammation were detected (Fig. 6B, upper image panel).

In dentin caries, a non-significant to weak decrease of EMILIN-1 and -2 was detected in the odontoblasts, in their processes, and in the ECM of pre-dentin as well as of the dental pulp (Fig. 6C). In contrast, dentin caries induced a significant increase in the signal intensity for EMILIN-3, which was particularly strong in odontoblast processes within tertiary dentin (Fig. 6C).

## Discussion

EMILINs are extracellular glycoproteins which fulfil regulatory functions in cell migration, differentiation, and proliferation by either binding to cell surface receptors such as integrins or directly modulating the activity of TGF-β, Wnt, and Hedgehog ligands^39–41^. Recently, we showed that EMILIN-1, -2, and -3 are targeted to FMF^36,37^ which are known to serve as architectural platforms to present and sequester growth factors of the TGF-β superfamily. This implicates that EMILINs endow cellular microenvironments with specialized functional and architectural properties to fine tune the bioavailability of growth factors in a contextual manner. Growth factor bioavailability is of fundamental interest for biologically oriented dental treatments^14,16^. Therefore, we were interested in investigating the expression and tissue localisation of EMILINs in the dentin-pulp complex. In this study, we report for the first time data on the spatio-temporal localization of all three EMILINs in the pulp of developing, adult, and carious teeth.

As previously shown in other tissues^31,42^ including the dental system, EMILINs have both overlapping and site specific distributions, with EMILIN-3 showing the most restricted expression. Interestingly, here we found that EMILIN-1 and -2, but not EMILIN-3, are present in the dental BM separating ameloblasts from odontoblasts. Furthermore, triple immunofluorescence stainings employing confocal immunofluorescence microscopy showed that EMILIN-1 and -2 are co-localized with laminin-γ1, nidogen-1, fibrillin-1, fibulin-1, and -2 within the DEJ and in the mesenchyme of the pulp horn. To our knowledge, this is the first report about the presence of EMILINs in these BM derived structures. In skin we previously demonstrated that EMILIN-1 co-localizes with FMF and EMILIN-1 positive fibers which insert perpendicularly into the BM of the dermal-epidermal junction^36^. Interestingly, it was reported that EMILIN-1 also forms protrusions reaching beyond the BM to make contact with basal keratinocytes^41^ and thereby controlling keratinocyte proliferation and differentiation via integrin binding. Our data suggest a similar regulatory function of EMILIN-1 and -2 on ameloblast behaviour within the DEJ. In line with our findings, mRNA and protein expression of fibulin-1 and -2 were previously detected at epithelial-mesenchymal interaction sites in two tissues of the developing embryo, the developing tooth and hair follicles^38^. Fibulin-1 and -2 were both found within BMs of the skin or endothelium of arteries^43,44^, where they are well anchored through interactions with BM proteins such as laminin 332 and nidogen-1, and -2^45^. Fibrillin-1 has been found in the BM in skin^46^ but could in this study for the first time be identified within the DEJ. Fibulin-2 was previously shown to interact with fibrillin-1 in BM regions, like in the kidney glomerulus and at the dermal–epidermal junction in skin^47^. Our pulldown assays showed for the first time that EMILIN-1 and -2 specifically interact with fibulin-2, suggesting that targeting and anchorage of EMILIN-1 and -2 to the DEJ is facilitated through specific interactions with fibulin-2 and fibrillin-1. Similarly, recruitment of EMILIN-1 to the dermal-epidermal BM of the hair follicle may take place by the same interactions, where EMILIN-1, fibulin-2, and fibrillin-1 form a mutual basket-shaped network around the hair bulb with EMILIN-1 forming protrusions toward the keratinocytes in the follicle bulb^41^.

Our localization studies showed that EMILIN-1, -2, -3 are all present in the condensed mesenchyme of the dental papilla and that EMILIN-1 and EMILIN-3 are also found in the adjacent mesenchymal tissues around the placode. EMILINs may not only play a role in ameloblast adhesion similar to collagens IV, VII, XVII, or laminin 332, but also in differentiation by modulating the bioavailability of growth factors. EMILIN-1 and -3 have both been described as extracellular inhibitors of TGF-β1, -2, and -3 signalling^31,32^, however it is still not clear if under physiological conditions EMILINs exert their activity as TGF-β inhibitors directly on the small latent complex or through an interaction with the large latent TGF-β complex and its components. Indeed EMILINs may influence the ECM targeting of LTBPs which serve as carriers of TGF-β forming the LLC. Previously, it was found that fibulin-2 competes with LTBP-1 for the same binding site on FMF^18^. Proper targeting of LTBPs to FMF is thought to be required for LLC stability to keep TGF-β in a latent state^17^. A corollary to that is that FMF deficiency leads to aberrant activation of TGF-β leading to imbalanced homeostasis in multiple connective tissues as observed in Marfan syndrome. Therefore, it is plausible that EMILINs by interacting with fibulin-2 may have a regulatory function in this process and thereby impact the ECM deposition and bioavailability of TGF-β.

Interestingly, EMILIN-3 was not only highly and specifically expressed in the early embryonic stages of tooth development, but also in the incisor mesenchymal stem cell niche with a distribution pattern that is resembling the one found in postnatal skin^37^, where EMILIN-3 is exclusively found in a narrow portion of the hair follicle bulge that lodges neural cells and that receives hedgehog signals. Intriguingly, both incisor and hair stem cells are known to be regulated by nerve-derived sonic hedgehog^48,49^ and EMILIN-3 has been reported to modulate hedgehog signalling in vivo in zebrafish embryos^34^. Altogether, this evidence points to a possible function of EMILIN-3 in fine tuning hedgehog signals directed to the incisor mesenchymal stem cells. Finally, our localization studies in human samples not only confirmed that all three EMILINs are found in the dental pulp, with patterns that mirror the murine ones, but also showed novel relevant findings: first, EMILIN-3, that showed only a weak staining in healthy conditions, was the only EMILIN member to be strongly upregulated in the odontoblast layer of carious molars. Under physiological conditions, the odontoblasts of the healthy adult human teeth form the secondary dentin matrix, while the odontoblasts of carious human teeth form the reactionary tertiary dentin matrix. Because EMILIN-1 and -2 were highly expressed in healthy odontoblasts, it can be assumed that EMILIN-1 and -2 may be involved in the regulation of secondary dentine matrix formation. In odontoblasts inflamed by carious lesion, a high expression of EMILIN-3 was found. This suggests that caries may induce a strong expression of EMILIN-3 in odontoblasts similar to the embryonic stages to regulate the formation of the reactionary tertiary dentin matrix.

Moreover, our co-localization studies demonstrated for the first time that EMILIN-3 was not only expressed by odontoblasts, but that the protein was also found in CD68 and HLA-DR positive cells. A role of EMILIN-3 in immunity has not yet been investigated, however, a recent gene expression study reported EMILIN-1 and EMILIN-2 as two of the most highly expressed glycoproteins in human macrophages during wound healing^50^. A previous study had already shown that EMILIN-1 and EMILIN-2 are strongly upregulated in the wound bed^36^. The subcellular localization of EMILIN-3 in CD68 and HLA-DR positive cells may indicate a role of EMILIN-3 in the differentiation of macrophages and/or dendritic cells. However, more studies are required to specifically address if EMILIN-3 may play a role during inflammatory and healing processes.

In conclusion we show for the first time that EMILINs are highly expressed in the dentin-pulp complex and that their distribution pattern is changing in dentin development and in dentin caries. The known function of EMILINs in growth factor signalling such as in the TGF-β, hedgehog, or apoptotic pathway makes them interesting targets for future research and new therapeutic approaches.

## Materials and methods

### Antibodies, recombinant proteins, expression plasmids

A rat monoclonal antibody against mouse EMILIN-1 (clone 1007C11A8)^40^ was used. Rabbit and guinea pig affinity-purified antibodies for EMILIN-2 and EMILIN-3 were already described^36,37^. Furthermore, anti-laminin-γ1^51^ (used at 1:1000), anti-nidogen-1 (gift from Mats Paulsson, University of Cologne; used at 1:1000), anti-CD68 (eBioscience, San Diego, CA; used at 1:2000), and anti-HLA-DR (eBioscience; used at 1:2000) primary antibodies were used. Affinity purified rabbit polyclonal antibodies directed against human fibrillin-1 (pAb9543), fibulin-1, and fibulin-2 were a kind gift from Takako Sasaki (Oita University, Japan). Recombinant murine C-terminally 2xStrepII tagged full length fibulin-1, and -2, as well as nidogen-1 were overexpressed, and purified as previously described^52^. Coding regions for full length murine EMILIN-1, -2, -3 were cloned with a C-terminally placed HA-tag into the pCS2 + vector as previously described^31^.

### Confocal immunofluorescence microscopy of mouse teeth

Dissected mouse embryo heads were embedded in OCT Compound (Sakura, Tokyo, Japan) and cryo-sectioned. Sections were fixed in ice-cold methanol/ acetone, blocked in a PBS containing 1% bovine serum albumin, and subsequently incubated with primary (rat anti-EMILIN-1 (1:500 dilution) (clone 1007C11A8)^40^, guinea pig anti-EMILIN-2 (1:1000 dilution), rabbit anti-EMILIN-3 (1:1000 dilution), and secondary Alexa dye conjugated antibodies (Thermo Fisher Scientific, Schwerte, Germany) diluted in the blocking solution. Cell nuclei were either stained with DAPI or DRAQ5 (both Thermo Fisher Scientific, Schwerte, Germany). Pictures were acquired with a Leica SP5 confocal laser microscope and processed with ImageJ software.

### Immunoblotting and in vitro binding assay

The lower first molar teeth of newborn C57BL/6N mice were removed, homogenized and 6 M guanidine hydrochloride extracts were prepared. SDS PAGE and immunoblotting was performed by using polyclonal antibodies raised against EMILIN-1, -2, and -3. In vitro binding assays were performed as previously described^30^. Briefly, cells were transfected with plasmids for EMILIN-1, -2, and -3 with a C-terminal HA tag. After transfection, cells were grown for 5 more days in serum free DMEM GlutaMAX medium (Invitrogen, Carlsbad, CA). Media were then collected and mixed with recombinant Strep-tagged fibulin-1 and fibulin-2 proteins gently shaking for 2 h at 4 °C. The mixtures were then subjected to precipitation with Strep-Tactin Sepharose (IBA, Goettingen Germany). Precipitated material was washed three times with the washing buffer (50 mM Tris–HCl, pH 7.5, 150 mM NaCl, 2 mM EDTA, 1% Triton X-100, and protease inhibitors) and proteins were resolved by SDS-PAGE and analyzed by western blotting using the following antibodies: monoclonal anti-HA and monoclonal anti-Strep antibodies (Sigma Aldrich, both used 1:1000).

### Immunohistochemistry of human molars

Healthy and inflamed third molars were obtained from patients who had undergone orthodontic extraction treatment. The age of the patients was between 17–26 years. No caries could be detected in asymptomatic healthy molars by radiography. Dentin and deep dentin caries of the carious molars was assessed by radiography. Molars with dentin caries were clinical characterized by reason-dependent painful stimuli, while deep dentin caries was clinically associated with spontaneous and/ or percussive persistent pain^53^. Histopathological diagnosis of healthy and carious molars was carried out by Haematoxylin and Eosin (H&E) staining. Three healthy and three carious molars (chronic dentin caries) from six different patients were selected for immunohistochemical experiments. Immunohistochemistry (IHC) was performed as previously described^53^ using guinea pig (anti-EMILIN-1 and -2, 1:1000) and rabbit (anti-EMILIN-3, 1:1000) primary antibodies. Biotinylated goat anti-guinea pig IgG, goat anti-rabbit IgG, normal goat serum (NGS), and Vectastain-ABC Kit were purchased from Vector Laboratories (Vector Laboratories, Burlingame, CA, USA). Pictures were acquired with a Zeiss Axioscope 2 microscope and analyzed by ImageJ.

### Statistical analysis

Data are expressed as mean ± SD. Statistical analyses were performed using GraphPad Prism software and the significance of differences between groups was determined by applying an unpaired two-tailed Student’s test. Values of P ≤ 0.05 were considered significant.

### Ethics statement

All research on human tissues was performed in accordance with local regulations, and was approved by the Human Ethics Committee of the Heinrich-Heine-University Düsseldorf (Nr.: 2,980). Informed consent was obtained from all participating donors who agreed to have materials examined for research purposes. Murine tissue isolation was carried out in strict accordance with the German federal law on animal welfare, and protocols for breeding and euthanasia of C57BL/6N mice were approved by the animal safety committee at the Medical Faculty, of the University of Cologne. Isolation of mouse embryos was approved by the North Rhine-Westphalian State Office for Environment, Health and Consumer Protection (2014.A224).

## Supplementary information

Supplementary figure 1

Supplementary figure 2

Supplementary figure 3

Supplementary figure 4

## References

[1] Thesleff I (2003). Epithelial-mesenchymal signalling regulating tooth morphogenesis. J. Cell Sci..

[2] Fukumoto S (2004). Ameloblastin is a cell adhesion molecule required for maintaining the differentiation state of ameloblasts. J. Cell Biol..

[3] Robinson C, Brookes SJ, Shore RC, Kirkham J (1998). The developing enamel matrix: nature and function. Eur. J. Oral Sci..

[4] Butler WT (1998). Dentin matrix proteins. Eur. J. Oral Sci..

[5] McGuire JD, Walker MP, Dusevich V, Wang Y, Gorski JP (2014). Enamel organic matrix: potential structural role in enamel and relationship to residual basement membrane constituents at the dentin enamel junction. Connect. Tissue Res..

[6] Tziafas D, Kodonas K (2010). Differentiation potential of dental papilla, dental pulp, and apical papilla progenitor cells. J. Endod..

[7] Tatullo M, Marrelli M, Shakesheff KM, White LJ (2015). Dental pulp stem cells: function, isolation and applications in regenerative medicine. J. Tissue Eng. Regen. Med..

[8] Ravindran S, Zhang Y, Huang C-C, George A (2013). Odontogenic induction of dental stem cells by extracellular matrix-inspired three-dimensional scaffold. Tissue Eng. Part A.

[9] Ravindran S, Huang C-C, George A (2014). Extracellular matrix of dental pulp stem cells: applications in pulp tissue engineering using somatic MSCs. Front. Physiol..

[10] Cox TR, Erler JT (2011). Remodeling and homeostasis of the extracellular matrix: implications for fibrotic diseases and cancer. Dis. Model. Mech..

[11] Lu, P., Takai, K., Weaver, V. M. & Werb, Z. Extracellular matrix degradation and remodeling in development and disease. *Cold Spring Harb. Perspect. Biol.* **3**, (2011). 10.1101/cshperspect.a005058 PMC3225943 21917992

[12] Sengle G, Sakai LY (2015). The fibrillin microfibril scaffold: A niche for growth factors and mechanosensation?. Matrix Biol..

[13] Wohl AP, Troilo H, Collins RF, Baldock C, Sengle G (2016). Extracellular regulation of bone morphogenetic protein activity by the microfibril component fibrillin-1. J. Biol. Chem..

[14] Oka S (2007). Cell autonomous requirement for TGF-β signaling during odontoblast differentiation and dentin matrix formation. Mech. Dev..

[15] Bègue-Kirn C (1992). Effects of dentin proteins, transforming growth factor beta 1 (TGF beta 1) and bone morphogenetic protein 2 (BMP2) on the differentiation of odontoblast in vitro. Int. J. Dev. Biol..

[16] Laurent P, Camps J, About I (2012). BiodentineTM induces TGF-β1 release from human pulp cells and early dental pulp mineralization. Int. Endod. J..

[17] Isogai Z (2003). Latent transforming growth factor β-binding protein 1 interacts with fibrillin and is a microfibril-associated protein. J. Biol. Chem..

[18] Ono RN (2009). Latent transforming growth factor β-binding proteins and fibulins compete for fibrillin-1 and exhibit exquisite specificities in binding sites. J. Biol. Chem..

[19] Sengle G (2008). Targeting of bone morphogenetic protein growth factor complexes to fibrillin. J. Biol. Chem..

[20] Sengle G, Ono RN, Sasaki T, Sakai LY (2011). Prodomains of transforming growth factor β (TGFβ) superfamily members specify different functions: extracellular matrix interactions and growth factor bioavailability. J. Biol. Chem..

[21] Horiguchi M (2009). Fibulin-4 conducts proper elastogenesis via interaction with cross-linking enzyme lysyl oxidase. Proc. Natl. Acad. Sci..

[22] El-Hallous E (2007). Fibrillin-1 interactions with fibulins depend on the first hybrid domain and provide an adaptor function to tropoelastin. J. Biol. Chem..

[23] Linde, A. Session II: Cells and Extracellular Matrices of the Dental Pulp—C.T. Hanks, Chairman: The Extracellular Matrix of the Dental Pulp and Dentin. *J. Dent. Res.* **64**, 523–529 (1985). 10.1177/002203458506400405 3857252

[24] Shuttleworth CA, Berry L, Kielty CM (1992). Microfibrillar components in dental pulp: presence of both type VI collagen- and fibrillin-containing microfibrils. Arch. Oral Biol..

[25] Liu J (2007). Matrix and TGF-β-related gene expression during human dental pulp stem cell (DPSC) mineralization. Vitro Cell. Dev. Biol. Anim..

[26] Kim J-H (2013). The role of lysyl oxidase-like 2 in the odontogenic differentiation of human dental pulp stem cells. Mol. Cells.

[27] Yoshiba N (2012). Expressional alterations of fibrillin-1 during wound healing of human dental pulp. J. Endod..

[28] De Coster PJ, Martens LC, De Paepe A (2004). Orofacial manifestations of congenital fibrillin deficiency: pathogenesis and clinical diagnostics. Pediatr. Dent..

[29] Sasaki T (2016). Loss of fibulin-4 results in abnormal collagen fibril assembly in bone, caused by impaired lysyl oxidase processing and collagen cross-linking. Matrix Biol..

[30] Schiavinato A (2017). Fibulin-4 deposition requires EMILIN-1 in the extracellular matrix of osteoblasts. Sci. Rep..

[31] Schiavinato A (2012). EMILIN-3, peculiar member of elastin microfibril interface-located protein (EMILIN) family, has distinct expression pattern, forms oligomeric assemblies, and serves as transforming growth factor β (TGF-β) antagonist. J. Biol. Chem..

[32] Zacchigna L (2006). Emilin1 links TGF-β maturation to blood pressure homeostasis. Cell.

[33] Marastoni S (2014). EMILIN2 down-modulates the Wnt signalling pathway and suppresses breast cancer cell growth and migration. J. Pathol..

[34] Corallo D (2013). Emilin3 is required for notochord sheath integrity and interacts with Scube2 to regulate notochord-derived Hedgehog signals. Development.

[35] Nakatomi Y, Tsuruga E, Nakashima K, Sawa Y, Ishikawa H (2011). EMILIN-1 regulates the amount of oxytalan fiber formation in periodontal ligaments in vitro. Connect. Tissue Res..

[36] Schiavinato A (2016). Targeting of EMILIN-1 and EMILIN-2 to fibrillin microfibrils facilitates their incorporation into the extracellular matrix. J. Invest. Dermatol..

[37] Corallo, D. *et al.* EMILIN3, an extracellular matrix molecule with restricted distribution in skin. *Exp. Dermatol.* 435–438 (2016). 10.1111/exd.13254 27892605

[38] Zhang H-Y, Timpl R, Sasaki T, Chu M-L, Ekblom P (1996). Fibulin-1 and fibulin-2 expression during organogenesis in the developing mouse embryo. Dev. Dyn..

[39] Spessotto P (2006). EMILIN1 represents a major stromal element determining human trophoblast invasion of the uterine wall. J. Cell Sci..

[40] Spessotto P (2003). β1 Integrin-dependent cell adhesion to EMILIN-1 is mediated by the gC1q domain. J. Biol. Chem..

[41] Danussi C (2011). EMILIN1–α4/α9 integrin interaction inhibits dermal fibroblast and keratinocyte proliferation. J. Cell Biol..

[42] Braghetta P (2004). Overlapping, complementary and site-specific expression pattern of genes of the EMILIN/Multimerin family. Matrix Biol..

[43] Longmate WM (2014). Reduced fibulin-2 contributes to loss of basement membrane integrity and skin blistering in mice lacking integrin α3β1 in the epidermis. J. Invest. Dermatol..

[44] Sicot F-X (2008). Fibulin-2 is dispensable for mouse development and elastic fiber formation. Mol. Cell. Biol..

[45] Timpl R, Sasaki T, Kostka G, Chu M-L (2003). Fibulins: a versatile family of extracellular matrix proteins. Nat. Rev. Mol. Cell Biol..

[46] Tsuji T (1980). Elastic fibres in the dermal papilla. Br. J. Dermatol..

[47] Reinhardt DP (1996). Fibrillin-1 and fibulin-2 interact and are colocalized in some tissues. J. Biol. Chem..

[48] Zhao H (2014). Secretion of Shh by a neurovascular bundle niche supports mesenchymal stem cell homeostasis in the adult mouse incisor. Cell Stem Cell.

[49] Brownell I, Guevara E, Bai CB, Loomis CA, Joyner AL (2011). Nerve-derived Sonic hedgehog defines a niche for hair follicle stem cells capable of becoming epidermal stem cells. Cell Stem Cell.

[50] Etich J (2019). Gene expression profiling of the extracellular matrix signature in macrophages of different activation status: relevance for skin wound healing. Int. J. Mol. Sci..

[51] Florea F (2014). Ex vivo pathogenicity of anti-laminin γ1 autoantibodies. Am. J. Pathol..

[52] Koch M (2006). Expression of type XXIII collagen mRNA and protein. J. Biol. Chem..

[53] Korkmaz Y (2011). Irreversible inflammation is associated with decreased levels of the α1-, β1-, and α2-subunits of sGC in human odontoblasts. J. Dent. Res..

