# Supplementary figures and images for: EMILIN proteins are novel extracellular constituents of the dentin-pulp complex
Source: Sci Rep. 2020 Sep 18;10:15320. doi: 10.1038/s41598-020-72123-2 (PMC7501263; doi:10.1038/s41598-020-72123-2)

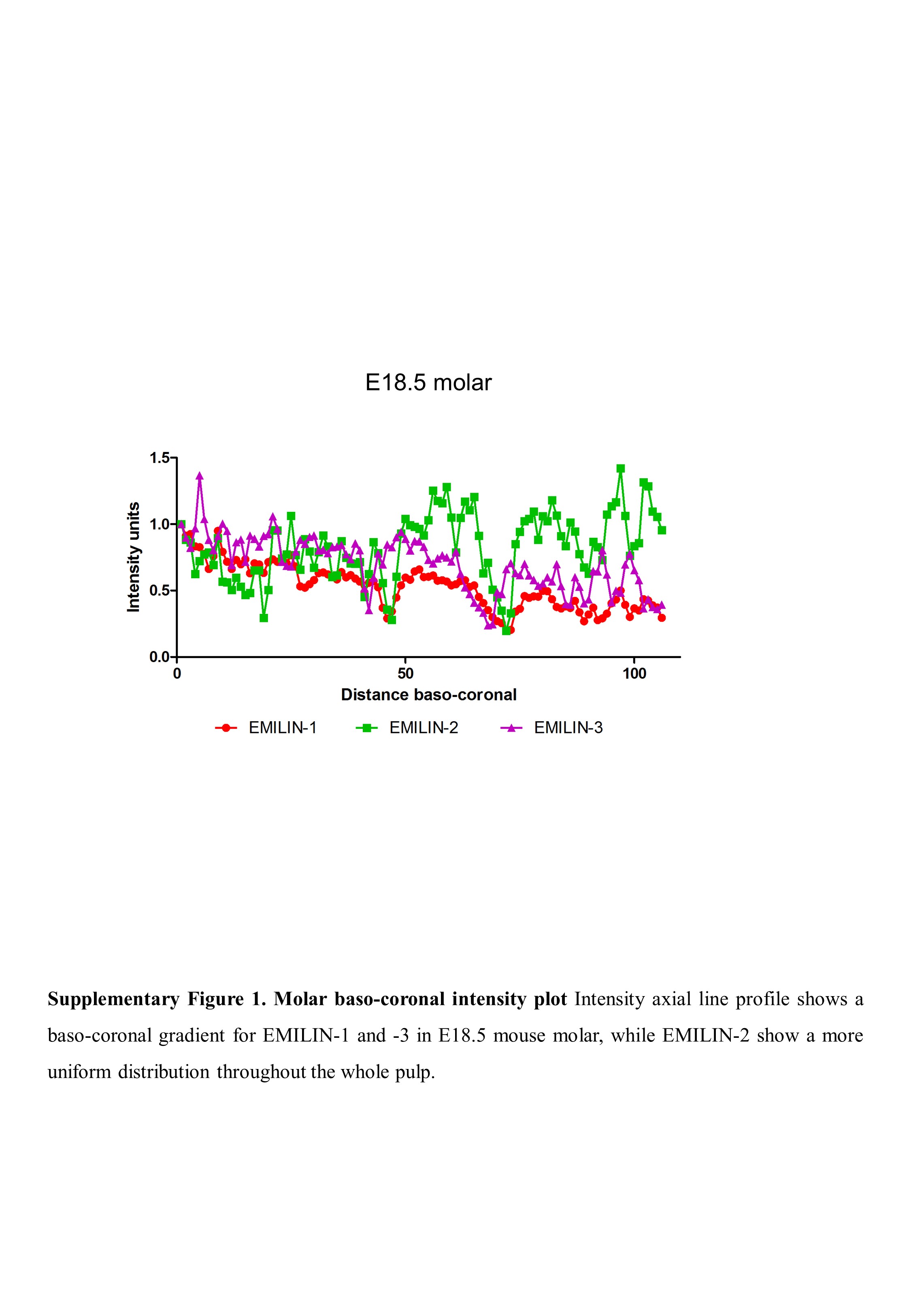

Supplement: Supplementary file 1 — Supplementary figure 1 [file 41598_2020_72123_MOESM1_ESM.jpg]

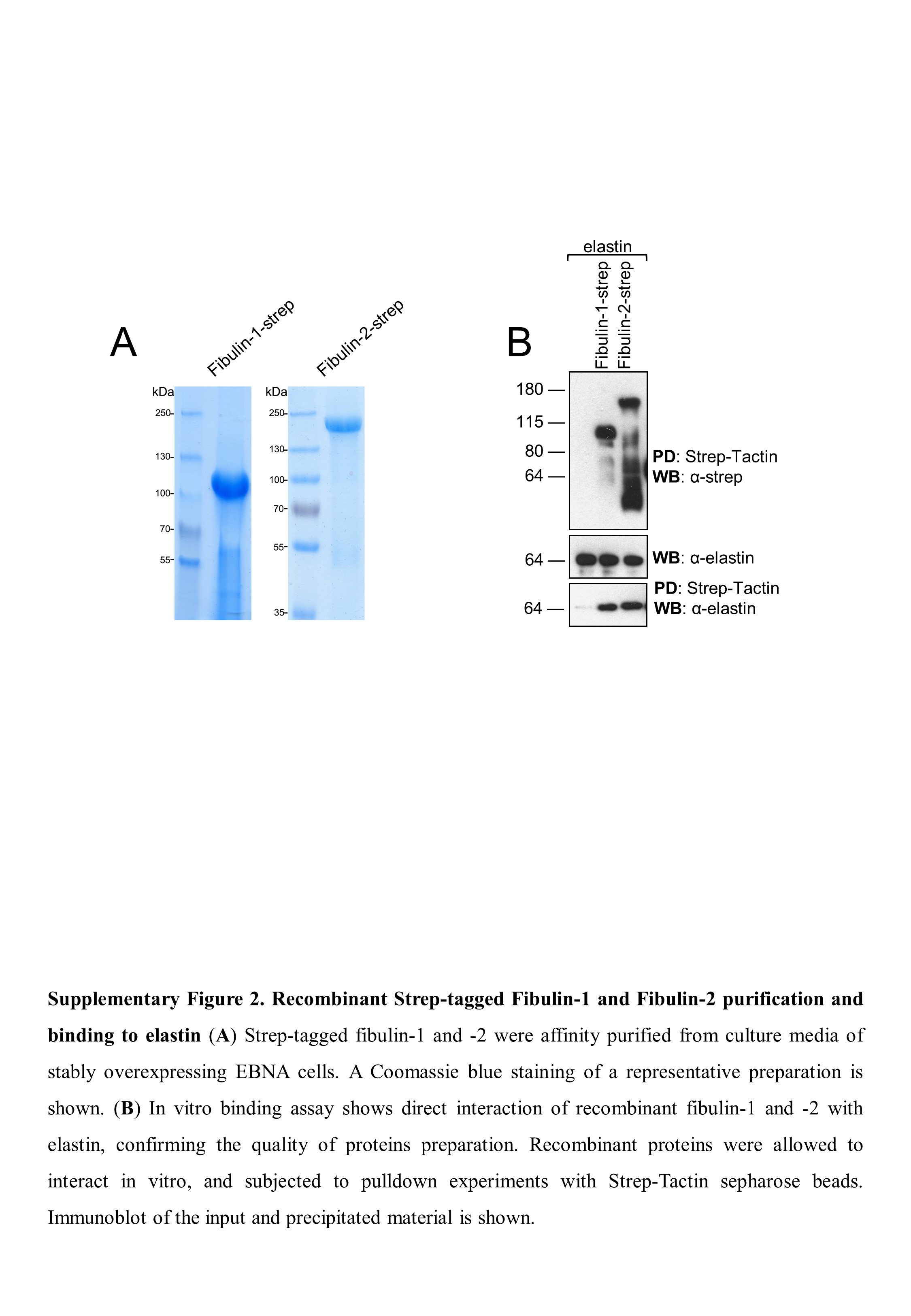

Supplement: Supplementary file 2 — Supplementary figure 2 [file 41598_2020_72123_MOESM2_ESM.jpg]

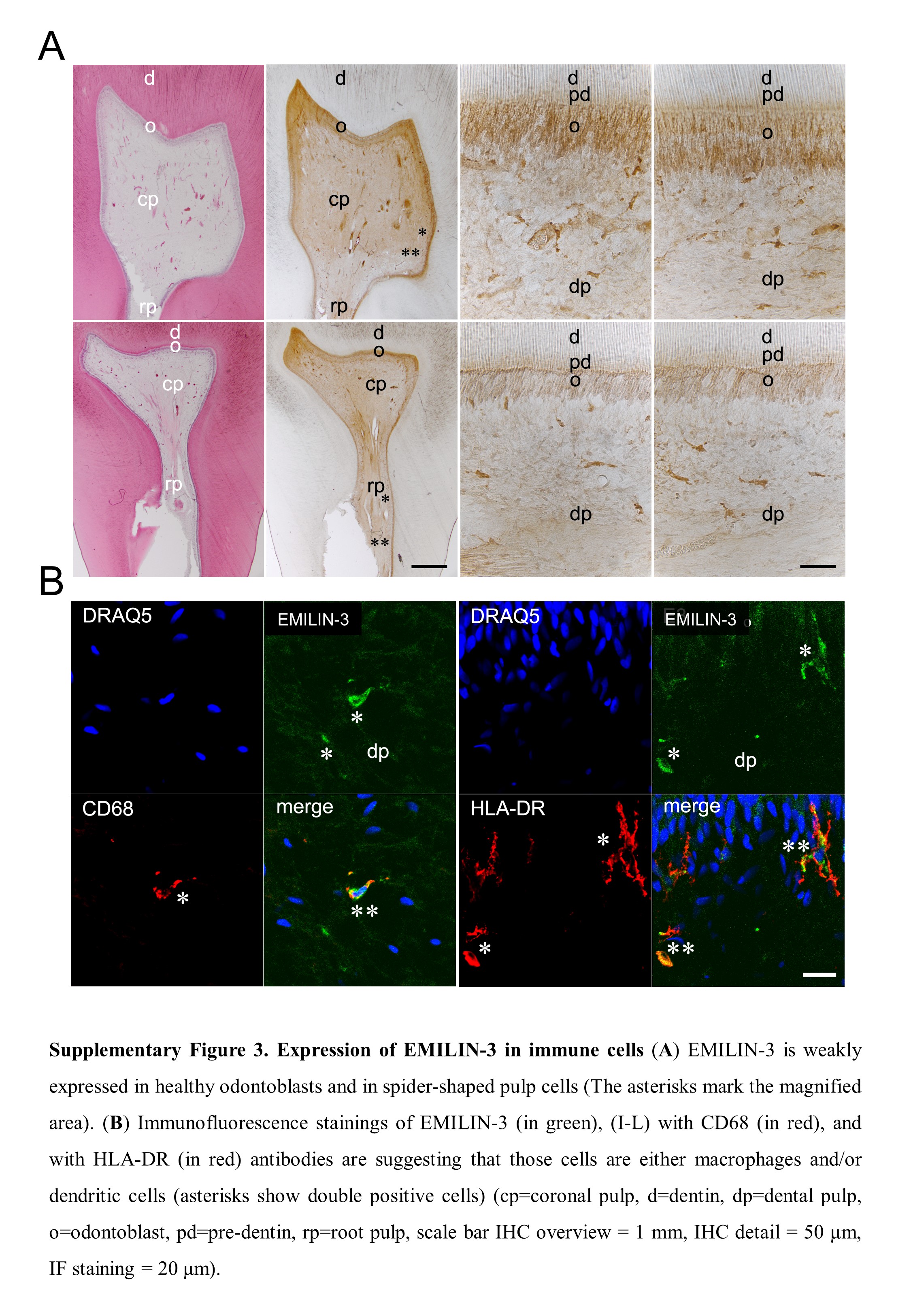

Supplement: Supplementary file 3 — Supplementary figure 3 [file 41598_2020_72123_MOESM3_ESM.jpg]

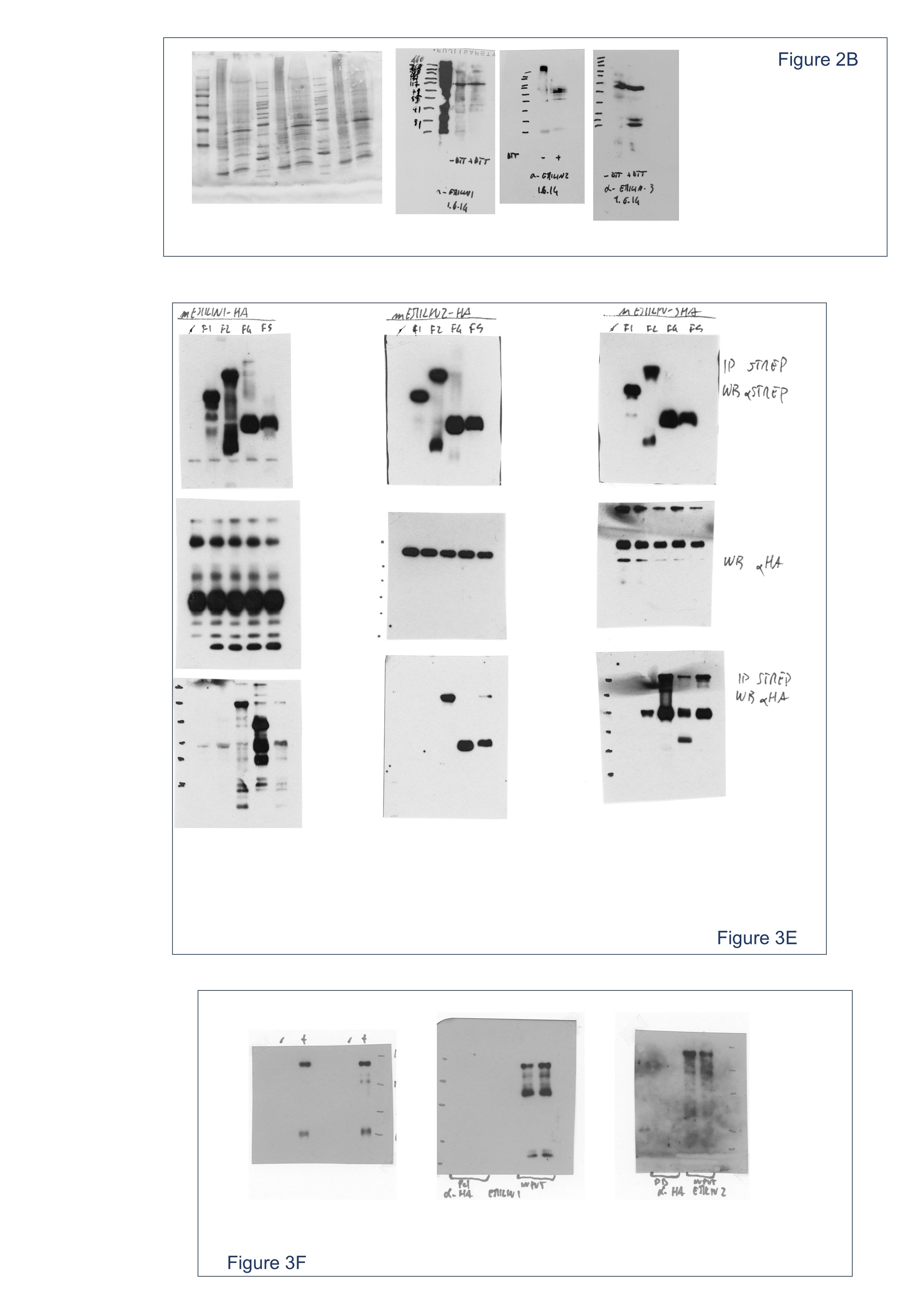

Supplement: Supplementary file 4 — Supplementary figure 4 [file 41598_2020_72123_MOESM4_ESM.jpg]
